# The predominant bacteria isolated from radicular cysts

**DOI:** 10.1186/1746-160X-9-25

**Published:** 2013-09-05

**Authors:** Mustafa Tek, Murat Metin, Ismail Sener, Cihan Bereket, Murat Tokac, Hakki O Kazancioglu, Seref Ezirganli

**Affiliations:** 1 Department of Oral and Maxillofacial Surgery, Faculty of Dentistry, Abant Izzet Baysal University, Bolu, Turkey; 2 Oral and Maxillofacial Surgen, Private Practitioner, Bursa, Turkey; 3 Department of Oral and Maxillofacial Surgery, Faculty of Dentistry, Ondokuz Mayis University, Samsun, Turkey; 4 Department of Medical Microbiology, Faculty of Medicine, Ondokuz Mayis University, Samsun, Turkey; 5 Department of Oral and Maxillofacial Surgery, Faculty of Dentistry, Bezmialem Vakif University, Istanbul, Turkey

**Keywords:** Radicular cyst, Bacteria, Predominant, Culture

## Abstract

**Purpose:**

To detect predominant bacteria associated with radicular cysts and discuss in light of the literature.

**Material and methods:**

Clinical materials were obtained from 35 radicular cysts by aspiration. Cultures were made from clinical materials by modern laboratory techniques, they underwent microbiologic analysis.

**Results:**

The following are microorganisms isolated from cultures: *Streptococcus milleri Group* (SMG) (23.8%) [Streptococcus constellatus (19.1%) and Streptococcus anginosus (4.7%)], *Streptococcus sanguis* (14.3%), *Streptococcus mitis* (4.7%), *Streptococcus cremoris* (4.7%), *Peptostreptococcus pevotii* (4.7%), *Prevotella buccae* (4.7%), *Prevotella intermedia* (4.7%), *Actinomyces meyeri* (4.7%), *Actinomyces viscosus* (4.7%), *Propionibacterium propionicum* (4.7%), *Bacteroides capillosus* (4.7%), *Staphylococcus hominis* (4.7%), *Rothia denticariosa* (4.7%), *Gemella haemolysans* (4.7%), and *Fusobacterium nucleatum* (4.7%).

**Conclusions:**

Results of this study demonstrated that radicular cysts show a great variety of anaerobic and facultative anaerobic bacterial flora. It was observed that all isolated microorganisms were the types commonly found in oral flora. Although no specific microorganism was found, Streptococcus spp. bacteria (47.5%) – especially SMG (23.8%) – were predominantly found in the microorganisms isolated. Furthermore, radicular cysts might be polymicrobial originated. Although radicular cyst is an inflammatory cyst, some radicular cyst fluids might be sterile.

## Introduction

Radicular cysts are chronic inflammatory lesions [1] and are considered to develop as a result of inflammation in periapical of tooth with pulp necrosis [2]. They are the most common seen odontogenic cysts (approximately 50% of all odontogenic cysts) [3,4]. Many studies have been performed to determine the relationship between the periapical lesions and the microorganisms [5-7].

Iatrou et al. [8] performed a study on infected jaw cysts and obtained cyst fluid samples and cultures, reporting that there were different bacteria in cyst fluids. However, a sufficient number of studies have not performed to illuminate microbiological content of radicular cysts.

Infections are formed in periapical region as a result of odontogenic diseases, and unsuccessful dental treatments, can sometimes become serious intracranial infections [9,10] and common head–neck infections [11-13]. Sometimes, serious and intractable infections can be formed as a result of an acute exacerbation in radicular cysts [14,15]. Obtaining further information on microorganisms causing infection will make the disease easier to treat. Although treatment of the radicular cyst is generally performed as a surgical procedure, use of antibiotics is sometimes considered to be supplementary, or to eliminate or limit the bacterial pathogens. Therefore, better knowledge of microorganisms will help improve the selection of antibiotics in oral and maxillofacial surgery practices.

The purpose of this study was to investigate the microbiological cultures created with sample materials obtained from radicular cysts through modern laboratory techniques, detect predominant bacteria associated with these lesions, and discuss findings in light of the literature.

## Materials and methods

We included patients who presented at Ondokuz Mayıs University, Faculty of Dentistry, Department of Oral and Maxillofacial Surgery Clinics. After examination and necessary tests, a total of 35 patients were randomly selected (25 males and 10 females) who were diagnosed with radicular cyst or residual cyst in maxilla and mandible, and clinically and radiologically delivered sample materials from cyst fluid to form microbiological cultures. The patients with antibiotics medication history within the last month were excluded from the study. All patients included in the study were asked to sign an informed consent form in accordance with Helsinki Declaration. The materials obtained from the patients were referred to SUVAM-TPL Bacteriology Department of Ondokuz Mayıs University for microbiological analysis.

### Obtaining sample material from cysts and transporting to laboratory

To obtain cyst fluid from 35 patients who were diagnosed with radicular cyst (23) or residual cysts (12), buccal mucosa was wiped with a sponge using an antiseptic solution Isosol® (OTC İstanbul İlaç Paz. Med. İtr. İth. İhr. Ltd. Şti., Istanbul, TURKEY) containing povidone–iodine of 10% and then irrigated with 20 ml sterile normal saline using a 10 ml disposable sterile injector. The buccal mucosa space was cleaned with a sterilized aspirator tip concurrently. Asepsis was ensured to prevent bacterial contamination during collection of clinical material. The area into which the cyst expanded further, where fluctuation was detected, or where the bone was resorbed the most, was wiped to dry with a sterilized dry sponge. Approximately 1–1.5 ml of cyst fluid was obtained from the said area with a 10 ml disposable sterile injector inserted into the cyst cavity. The air in the injector was deflated completely, and the tip of the injector was bent to prevent air intake. The clinical materials obtained were transported to SUVAM-TPL Bacteriology Department of Ondokuz Mayıs University within 20 minutes. For a final diagnosis of cysts, the cyst mucosa obtained after the enucleation or marsupialization of cysts was transported to the pathology laboratory in a bottle containing 10% formalin [8].

### Microbiologic laboratory procedures

After the sample materials in the injector were transported to the bacteriology laboratory, they were macroscopically checked by color, consistency, smell, and whether they contained sulfide granules. Blood agar was used to obtain aerobic cultures of clinical samples; Schaedler Agar with addition of vitamin K1, prepared daily, and broth (Oxoid LTD, Basingstoke, Hampshire, England) were used to obtain anaerobic cultures. Two different methods were used to have an anaerobic environment: The first one was closable 2.5-liter glass jars in which GENbag anaer (bioMérieux sa, France) used for 2.5 liter was placed to ensure an anaerobic environment. The second method was closable nylon bags in which bag-compatible GENbag anaer (bioMérieux sa, France) was placed to ensure an anaerobic environment. Strips impregnated with methylene blue (Merk KGaA, Germany) were used to checker whether anaerobic conditions were formed in the atmosphere of both systems.

Aerobic cultures were incubated at 37°C for 24–48 hours, and anaerobic cultures were incubated at 37°C for 7 days. The colonies grown in Schaedler agar, which were incubated anaerobic, were compared with the colonies in blood agar, which was kept in aerobic conditions.

If there were haze or malodor in the Schaedler broth, just the colonies grown in anaerobic conditions, as a result of passaging, suggested anaerobic bacteria, whereas bacteria grown in either of them suggested facultative anaerobic bacteria. The isolated bacteria were stained with Gram’s method. API 20 A panel (bioMérieux sa, France) and a computer system were used to identify the isolated bacteria [7]. The data obtained were evaluated statistically.

Review Board of Institute of Health Sciences in Ondokuz Mayis University approved this study. Reference number: (2004/ADÇ.119).

## Results

The average age of all patients included in the study was 36.4 ± 14.1 years; the average age of males was 38.2 ± 15.3 years, and the average age of females was 32.0 ± 10.0 years. The clinical materials of 35 patients with cyst (23 radicular cysts and 12 residual cysts) that were obtained and cultured were analyzed microbiologically by aerobic and anaerobic. The bacteria were isolated in 13 (37.1%) of all cases. Sixteen different types of microorganisms were derived from 13 cases. From each case with bacteria grown, 1–3 bacteria (mean 1.61) were derived.

The following were microorganisms isolated from cultures: *SMG* (23.8%) [*Streptococcus constellatus* (19.1%) and *Streptococcus anginosus* (4.7%)], *Streptococcus sanguis* (14.3%), *Streptococcus mitis* (4.7%), *Streptococcus cremoris* (4.7%), *Peptostreptococcus pevotii* (4.7%), *Prevotella buccae* (4.7%), *Prevotella intermedia* (4.7%), *Actinomyces meyeri* (4.7%), *Actinomyces viscosus* (*4*.*7*%), *Propionibacterium propionicum* (4.7%), *Bacteroides capillosus* (4.7%), *Staphylococcus hominis* (4.7%), *Rothia denticariosa* (4.7%), *Gemella haemolysans* (4.7%), and *Fusobacterium nucleatum* (4.7%).

Of microorganisms isolated from the cultures, 14 were facultative anaerobic and 7 were obligate anaerobic bacteria. All facultative anaerobic bacteria were Gram positive; of obligate anaerobic bacteria, 3 types were Gram positive and the other 4 types were Gram negative (Table 1).

**Table 1:** Bacteria isolated from radicular cysts

**Species of bacteria**	**Number of isolates**	**Gram stain**
**Facultative anaerobes, microaerophiles, and capnophiles**		
*Streptococcus constellatus* (SMG)*	4	Gram ( + )
*Streptococcus anginosus* (SMG)*	1	Gram ( + )
*Streptococcus sanguis*	3	Gram ( + )
*Streptococcus mitis*	1	Gram ( + )
*Streptococcus cremoris*	1	Gram ( + )
*Staphylococcus hominis*	1	Gram ( + )
*Rothia denticariosa*	1	Gram ( + )
*Actinomyces viscosus*	1	Gram ( + )
*Gemella haemolysans*	1	Gram ( + )
**Total Aerobic Bacteria**	**14**	
**Strict anaerobes**		
*Prevotella buccae*	1	Gram ( - )
*Prevotella intermedia*	1	Gram ( - )
*Propionibacterium propionicum*	1	Gram ( + )
*Bacteroides capillosus*	1	Gram ( - )
*Actinomyces meyeri*	1	Gram ( + )
*Peptostreptococcus pevotii*	1	Gram ( + )
*Fusobacterium nucleatum*	1	Gram ( - )
**Total Strict Anaerobic Bacteria**	**7**	

*SMG: *Streptococcus milleri Group.*

The status of isolated bacteria from the cultures are seen according to the localization of the cyst (Table 2), the properties of the cyst fluid (Table 3), and oral hygiene of the patients (Table 4).

**Table 2:** The relationship between the bacterial growing and the localization of radicular cysts

** Localization of cyst**	**Bacterial growing**		**Case number**
	**Positive**	**Negative**	
Maxilla	6	12	18
Mandible	7	10	17
**Case Number**	13	22	35*

*Total number of the samples used in this study.

**Table 3:** The relationship between the bacterial growing and the properties of cyst fluids

** Properties of cyst fluid**	**Bacterial growing**		**Case number**
	**Positive**	**Negative**	
Transparent colored fluid	-	9	9
Straw colored fluid	11	8	19
Whitish colored fluid	1	1	2
Straw colored viscous fluid	1	1	2
Brownish colored viscous fluid	-	3	3
**Case Number**	13	22	35*

*Total number of the samples used in this study.

**Table 4:** The relationship between the bacterial growing and the oral hygiene of patients

** Oral hygiene**	**Bacteria growing**		**Case number**
	**Positive**	**Negative**	
Well	1	2	3
Mild	6	14	20
Poor	6	6	12
**Case Number**	13	22	35*

*Total number of the samples used in this study.

The cyst mucosa obtained after surgical treatment of all patients diagnosed with radicular cyst or residual cyst, clinically and radiologically, was examined histopathologically and diagnoses were verified.

## Discussion

Radicular cysts arise from periapical granulomas formed on devital teeth. They are the most common lesions [16,17] among tooth-originated periapical lesions, apart from periapical abscess [16] and periapical granulomas [17]. In addition, radicular cysts are the most common among all jaw cysts [3,4]. Therefore, determination of microorganisms associated with radicular cyst will ensure more conscious treatment of infections that might occur in the jaw.

The biggest problem with microbiological studies relating to the lesions in the periapical region is contamination with the microbial flora of surrounding tissue [5,7,8]. To avoid bacterial contamination in our study, the oral mucosa and teeth were wiped with povidone–iodine 10% prior to the aspiration of cyst fluid. The aspiration area was then irrigated with sterilized normal saline and wiped with a sterile sponge.

Antiseptic mouthwashes [18] and antibiotics is used to reduce the number of microorganisms [19]. In the case of administration of antibiotics, it is reported that antibiotics penetrated into the periapical lesions [20] and radicular cysts [21] in jaws. Iatrou et al. [8] isolated bacteria in 25 patients (67.6%) of 37 patients who had been on antibiotic medication before collecting samples from infected jaw cysts (1–6 days prior). The bacteria were grown in 12 patients (70%) of 17 patients who had not taken any antibiotics within the prior month. No statistical significant difference was found between the two groups; however, the patients who had taken any antibiotics within the prior month or had been on antibacterial medication were not included in our study because of the possibility of affecting the results.

Additionally, the results of this study showed that bacterial growing was higher in the cysts of mandible (53.85%) when compared with the cysts of maxilla (46.15%). We found that the transparent colored fluids (totally 9 cases) and the brownish colored viscous fluids (totally 3 cases) were been sterile and the bacteria was isolated from 57.89% of all straw colored fluids (totally 19 cases), 50% of the whitish colored fluids (totally 2 cases), and 50% the straw colored viscous fluids (totally 2 cases). Furthermore, we also found that positive culture was been in 33.33% of the patients who have well oral hygiene (totally 3 patients), in 30% of mild oral hygiene (totally 20 patients), and in 50% of poor oral hygiene (totally 12 patients). According to these results, oral hygiene, localization of cyst, and color of cyst fluid might be affect on bacterial growing.

In a study performed by Iatrou et al. [8] on infected jaw cysts – radicular, residual, dentigerous, globulomaxillary, incisive canal, and keratocyst – bacteria were isolated in 78.6% of microbiological cultures in 54 infected cyst fluid samples. The patients in our study were selected randomly and not categorized by infection. The bacteria were isolated in 13 (37.1%) of 35 cysts (23 radicular, 12 residual). This difference in results might be the selection of patients.

It is reported in the literature that the type of anaerobic bacteria were higher than the type of aerobe and facultative anaerobic bacteria when reviewing microbiological analysis of dentoalveolar infections [22-26]. Iatrou et al. [8] determined in their study performed on infected jaw cysts that a large part of isolated bacteria (89.2%) was anaerobic bacteria. Aerobe and facultative anaerobic bacteria growth were seen in 10.8% of the cases. In their study, a large part of isolated bacteria were found to be of those in normal oral microflora. For all that, in this study, of the microorganisms isolated from sample cultures, 14 (66.7%) were facultative anaerobic and 7 (33.3%) were obligate anaerobic bacteria. The isolated bacteria were determined to be the elements of normal oral flora.

In their study, Iatrou et al. [8] obtained a total of 84 types of bacteria in infected jaw cysts in which bacterial growth was observed. From each case with bacteria positive, 1–4 different colonies of bacteria were isolated. The greater part of isolated bacteria was Gram positive (59.5%) and the others were Gram negative (40.5%). In our study, 16 different types of bacteria were derived from 13 cases. Of the bacteria positive cases, 6 were polymicrobial and 7 were single types of bacteria. From each case with bacteria positive, 1–3 (mean 1.6) different types of bacteria were isolated. A large part of all isolated bacteria was Gram positive (81%), and the others were Gram negative (19%). There is a lot of diversity in the organisms found, the reason of these results may be caused by oral cavity organisms.

SMG has also been reported to be significant pathogens [27]. However, these pathogens are part of the normal flora of human oral cavity [28] and other mucous membranes [29-32]. SMG is not included in the approved lists of bacterial names, and is composed of three distinct species: *Streptococcus anginosus*, *Streptococcus constellatus*, and *Streptococcus intermedius*[33]. Further, SMG describes a group of anaerobic or microaerophilic streptococci. These organisms are frequently isolated from purulent infections of the head and neck region [34] and internal organs including: brain [35], liver [36], and lungs [37]; and from cases of endocarditis [38] and meningitis [39]. They might cause infective endocarditis, such as streptococci of other mucous membranes; however, they have also been recurrently associated with severe suppurative infections, unlike streptococci of other mucous membranes [32].

SMG bacteria are important pathogenic microorganisms for infections and abscess of the orofacial region, according to literature survey. In the present study, SMG (23.8%) and *Streptococcus sanguis* (14.28%) were predominantly isolated from sample cultures. In addition, facultative anaerobic bacteria found to be higher in bacteria were isolated from cultures. Iatrou et al. [8] reported that the predominant group of bacteria isolated from infected jaw cysts was Gram-positive anaerobic coccus (36.9%). Hrvacanin et al. [40] analyzed radicular cyst fluid and often isolated ∝-*haemolytic streptococcus*, *Streptococcus pneumoniae*, *Staphylococcus epidermidis*, *and Streptococcus group B* type of bacteria.

Actinomyces are Gram-positive, non-acid fast, anaerobic or microaerophilic filamentous branched bacteria that are very difficult to grow in culture. In man, *Actinomyces* spp. bacteria may cause severe head and neck infections. The pathogenic *Actinomyces* most frequently isolated is *Actinomyces israelii*. *Actinomyces propionica*, *Actinomyces naeslundii*, *Actinomyces viscosus*, and *Actinomyces odontolyticus* are less common infections. These bacteria are all normal commensals of the human oral cavity [41-46]. *Actinomyces* spp. bacteria were not isolated by Iatrou et al. [8]. However, Hirsberg et al. [47] performed a study and analyzed periapical biopsy samples histologically. They found typical actinomycotic colonies in 1.8% of the samples. Additionally, Tseng at al. [15] reported the presence of *Actinomyces* spp. bacteria in the case of a radicular cyst. The type of *Actinomyces meyeri* and *Actinomyces viscosus* (9.52%) bacteria were isolated also in our study.

Additionally, radicular cyst is generally treated using surgical enucleation or/and marsupialization. If it becomes necessary to antibiotic therapy to help for the treatment of radicular cyst, the surgeon should consider the results of this study for the selection of antibiotic.

## Conclusion

Results of this study demonstrated that radicular cysts show a great variety of anaerobic and facultative anaerobic bacterial flora. It was observed that all isolated microorganisms were the types commonly found in oral flora. Although no specific microorganism was found, Streptococcus spp. bacteria (47.5%) – especially SMG (23.8%) – were predominantly found in the microorganisms isolated. Furthermore, radicular cysts might be polymicrobial originated. Although radicular cyst is an inflammatory cyst, some radicular cyst fluids might be sterile.

### Consent statement

All patients included in the study were asked to sign an informed consent form in accordance with Helsinki Declaration.

## Abbreviations

SMG: Streptococcus Milleri Group; Spp: Species.

## Competing interests

The authors declare that they have no competing interests.

## Authors’ contributions

MT and MM conceived of the study, and participated in its design and coordination and helped to draft the manuscript. MT, IS, and CB collected the data. MTo performed microbiological examination, analysed and interpreted the data. MT, HOK and SE wrote the manuscript. HOK and SE participated in its English editing. All authors read and approved the final manuscript.
